# Effect of conjugated linoleic acids from beef or industrial hydrogenation on growth and adipose tissue characteristics of rats

**DOI:** 10.1186/1743-7075-6-19

**Published:** 2009-04-22

**Authors:** Mao L He, Priya S Mir, Erasmus K Okine, Helen Napadajlo

**Affiliations:** 1 Agriculture and Agri-Food Canada, Lethbridge, Alberta, T1J 4B1, Canada; 2 Department of Agriculture Food and Nutritional Science, University of Alberta, Edmonton, Alberta, T6G 2P5, Canada

## Abstract

**Background:**

The conjugated linoleic acid (CLA) content of beef can be increased by supplementing appropriate beef cattle diets with vegetable oil or oil seed. Yet the effect of consumption of such beef on adipose tissue characteristics is unclear, thus the study was conducted to compare adipose tissue responses of rats to diets containing beef from steers either not provided or provided the oil supplements to alter CLA composition of the fat in muscle.

**Methods:**

Effects of feeding synthetic (industrial hydrogenation) CLA or CLA from beef on growth and adipose tissue responses of weanling, male, Wistar rats (n = 56; 14 per treatment diet) were investigated in a completely randomized design experiment. Diets were: control (CON) diet containing casein and soybean oil, synthetic CLA (SCLA) diet; where 1.69% synthetic CLA replaced soybean oil, two beef-diets; CONM and CLAM, containing freeze dried beef from steers either not fed or fed 14% sunflower seeds to increase CLA content of beef. Diets were isonitrogenous (20% protein) and isocaloric. Rat weights and *ad libitum *intakes were recorded every 2 wk. After 9 wk, rats were fasted for 24 h, blood sampled by heart puncture, sacrificed, tissue and organs were harvested and weights recorded. The adipose tissue responses with regard to cellularity and fatty acid compositions of retroperitoneal and inguinal adipose tissue were determined.

**Results:**

Body weights and gains were comparable, but organ weights as percent of body weight were greater for rats fed SCLA than CONM. Fasting blood glucose concentration was lower (p < 0.01) in rats fed SCLA than those fed CONM or CLAM. Retroperitoneal and inguinal fat weights, as percent of body weight were greater (p < 0.01) in rats fed CONM or CLAM than those fed CON or SCLA diets. Adipocyte numbers were least in retroperitoneal tissue of rats fed SCLA, while inguinal tissue cell density and total number were lower (p = 0.02) in rats fed CLAM (7.26 × 10^7 ^cells/g and 8.03 × 10^8 ^cells) than those fed CONM (28.88 × 10^7 ^cells/g and 32.05 × 10^8 ^cells, respectively).

**Conclusion:**

Study suggests that dietary CLA either as synthetic or high CLA-beef may alter adipose tissue characteristics by decreasing the number of adipocytes and by decreasing the size of the tissue.

## Background

The probable health benefits that could ensue from consumption of the biologically effective isomers of conjugated linoleic acids (CLA) has led to studies that could increase CLA isomers in foods and to documentation of the effects of consumption of the CLA isomers as synthetic compounds or as components in food. Among the health benefits of CLA consumption are the anti-carcinogenic effects [1-3] and probable anti-atherogenic effects [4,5], which appear to be related to the effects of the CLA isomers on lipid metabolism. The CLA trans (t)10, cis (c)12 isomer appears to inhibit lipogenesis in mouse cells [6,7] and human adipocytes [8,9], by affecting differentiation via gene expression [9-11]. The commonly occurring isomer, the CLA c9, t11 appears to be anti-proliferative to mouse adipocytes in vitro [8,9,12]. In vivo, total body fat was reduced when animals were fed a mixture of the two CLA isomers from synthetic sources [13-17]. Similar results were reported in some studies with human subjects [18,19].

The CLA c9, t11 is the major isomer that occurs in ruminant food products, such as milk, beef and mutton [20] and is produced either by the bacterium *Butyrivibrio fibrosolvens *in the rumen [21] or by desaturation of C18:1 t11 at the ninth carbon in muscle, liver or mammary gland [22]. However, CLA t10, c12 is largely a product of rumen fermentation [23] and both CLA isomers can accumulate in fat tissues, with the concentration being affected by the feed provided to the cattle [24]. The CLA content in meat of ruminants ranges from 1.7 to 8.5 mg CLA/g lipid [25,26], depending on species, breed and feeding regimes [26,27]. The CLA content of beef can be increased by supplementation of appropriate diets with sources of vegetable oil [27,28]. Although it is difficult to increase the bio-formed CLA in beef to levels such as to meet recommended intakes for humans, substantial increases can be achieved by feeding oils high in polyunsaturated fatty acids (PUFA) to cattle. Furthermore, studies indicate that substantially lower intakes of bio-formed CLA in foods could decrease incidence of cancer [29,30] or more effectively reduce proliferation of cancer cells *in vitro *than synthetic fatty acid mixtures, which are similar to beef fatty acid composition [31]. In a pilot study, rats consuming a high CLA beef, produced by feeding cattle a diet supplemented with sunflower oil, reduced adipocyte number in both inguinal (IG) and retroperitoneal (RP) fat pads relative to rats fed beef from animals not provided the oil substrate [17]. Similarly, even though the dietary concentrations of the CLA from beef containing diets were substantially lower than that provided as synthetic CLA, a reduction in the adipocyte number in the rat adipose tissues was observed and may be due to inhibition of proliferation of adipocytes by the bio-formed CLA.

Therefore, the present study was conducted to determine the effects on adipose tissue characteristics, of bio-formed CLA, as beef from steers fed sunflower seeds (SS) at 14% of diet dry matter [28], in rats and compared to those of rats fed beef from steers not provided the SS. Included also were diets with casein as a dietary protein without or with supplemental synthetic CLA derived from industrial hydrogenation of vegetable oil. The experiment was conducted using a completely randomized design, where the diets were provided at 20% protein, to control the dietary protein content, unlike the previous study where the meat replaced the casein [17]. The responses investigated were growth of the rats, relative weights of the tissues such as muscle, organs and adipose tissues to total body weight and the adipocytes in two adipose tissues were enumerated. The lipid content and fatty acid composition of the adipose tissues were also determined.

## Methods

### Animals and diets

A total of 56 weaned, male Wistar strain rats were obtained from Charles River, Canada (St. Constant, Quebec, Canada) and were housed individually in cages upon arrival, and provided rat feed pellets and water *ad libitum *for five days to allow them to adjust to the new surroundings. Rats were randomly allocated to four treatments (n = 14) by weight and maintained on a 12 h light:dark cycle at 22 ± 2°C and cared for according to the guidelines of the Canadian Council on Animal Care [32] and the experiment was conducted with the approval of the Institutional Animal Use Committee (Approval number – 0219).

The diets (Table 1) were: the control diet (CON) – composed of purified ingredients (AIN-93 diet) with casein as the protein source; the synthetic CLA diet (SCLA) – which was the CON diet where 1.69% of the soybean oil was replaced with an equivalent amount of synthetic CLA mixture; the control meat diet (CONM) – which employed freeze dried beef from animals not fed supplemental SS (control beef) and the CLA beef diet (CLAM) – which contained freeze dried beef from cattle fed 14% SS to elevate the CLA and PUFA contents [28] relative to the control beef. In the meat diets the beef from the two sources replaced the casein and soybean oil to the extent necessary to meet the protein and fat concentration of the diets. The synthetic CLA used in this study was a feed-grade CLA containing 60% CLA, of which 53% was CLA c9, t11 and 44% was the CLA t10, c12 isomer, and trace amounts of the other CLA (Bioriginal Food & Science Corp., Saskatoon, Canada).

**Table 1:** Dietary ingredients and nutrient composition

	Treatment diets			
	
	CON	SCLA	CONM	CLAM
**Ingredient (g/10 kg of diet)**				
L-cystine* cat # 101444	30.0	30.0	30.0	30.0
Corn starch* cat# 902956	5288.0	5277.0	5090.0	5001.0
Alphacel* cat # 900453	500.0	500.0	500.0	500.0
Vitamin mix* cat # 960402	100.0	100.0	100.0	100.0
Mineral mix* cat # 960400	351.0	351.0	351.0	351.0
Choline bitartrate* cat # 101384	25.0	25.0	25.0	25.0
Casein vitamin-free*cat #904520	1950.0	1950.0	1100.0	537.0
Sucrose* cat # 902978	1000.0	1000.0	1000.0	1000.0
Soybean oil* cat #104687	656.0	498.0	0.0	0.0
Synthetic CLA^^^	0.0	169.0	0.0	0.0
Control beef	0.0	0.0	1804.0	0.0
High CLA beef	0.0	0.0	0.0	2456.0
**Nutrient composition**				
DM %	93.5	93.7	94.0	94.0
Total lipid (%DM)	7.3	7.3	5.7	7.9
Total protein (%DM)	19.6	20.1	19.9	21.9
Gross energy (Kcal/g DM)	4.34	4.22	4.37	4.24

*purchased from MP BioMedicals with catalog number (cat #)

^^^purchased from Bioriginal Food and Sceince Corporation, Saskatoon – Contained 60% Conjugated linoleic acid (CLA) with 53%CLA cis (c)9, trans (t)11 and 44% CLA t10, c12 isomers.

Control beef – beef from steers not provided sunflower seeds (SS) during their production, with composition; 97% dry matter (DM), 52.6% protein and 38.8% lipids in DM.

High CLA Beef-beef from steers fed 14% SS during their production with composition; 95% dry matter (DM), 60.6% protein and 28.5% lipids in DM.

CON – Control diet, SCLA – diet with 1.69% synthetic CLA replacing and equivalent amount of the soybean oil, CONM – Diet containing control beef, CLAM – diet containing high CLA beef.

Body weight was recorded every two weeks, while feed was provided *ad libitum *and weighed in every second day. The residual feed and the rats were weighed on the same day. Eight weeks post initiation of the experiment, over the following two weeks, the animals were sacrificed after a 24 h feed withdrawal prior to the day of euthanasia. Each day, the heaviest rat fed from each diet, was sedated with isoflourine and blood was collected by heart puncture and then euthanized by carbon dioxide suffocation prior to processing for tissue collection.

### Blood and tissue sampling

Serum was harvested from trunk blood samples with a minimal lapse in time and used for determination of glucose, triglycerides and cholesterol concentrations. The fresh rat carcasses were weighed after decapitation. The heart, liver, kidney, inguinal and retroperitoneal fat pads and the soleus and gastrocnemius muscles from the right half of the carcass were dissected and weighed. Weights of the heart, liver, kidney and the tissues are reported as actual weight and as a percentage of the body weight in order to determine if the diets influenced development of particular organs. Fresh fat tissues were sampled and processed for determination of cell number and the remaining adipose tissue samples were stored at -20°C until analyses for fat content and fatty acid composition.

### Adipocyte enumeration in retroperitoneal and inguinal adipose tissues

Adipocytes in the fat pads were enumerated after fixing approximately 80 mg of fresh tissue in osmium tetroxide [33] after removal of blood cells, stromal vascular cells and pre-adipocytes. The fixed cells that passed through a 240 μmesh [34] were enumerated after appropriate dilutions [17]. The cells were enumerated using a particle counter (Coulter counter, Model ZBI, Coulter Electronics Inc. FL, USA) after standardizing the equipment with hematology controls (Para 12, Streck Laboratories, Inc., NE, USA).

### Fat extraction and fatty acids analysis

Lipids from fat tissues, from half the rats (n = 7; randomly selected), from each treatment were extracted according to the procedures of Jiang and coworkers [35] with modifications. Briefly, 15 mL of isopropanol was added to a test tube containing the tissue sample (1.0 g) and then homogenized (Polytron PT 10–35, Kinematic AG, Switzerland) at high speed for 15 sec. A further 10 mL of hexane was added and homogenized for 15 seconds. The homogenized mixture was filtered through Whatman filter paper. The above procedure was repeated twice with two, 10 mL portions of hexane-isopropanol (v:v; 10:15). Then 8 mL of aqueous sodium sulfate solution (66.8 g Na_2_SO_4_/L of distilled water) was added to the filtrate and thoroughly mixed. The test tube containing the solution was centrifuged at 1100 rpm (640 × g) for 10 min. The hexane layer was collected into pre-weighed test tubes, and evaporated under nitrogen completely to obtain the lipids. The residual fat with the tube was weighed to calculate the fat content of the sample.

A combined base/acid methylation was used to prepare the fatty acid methyl esters [36]. Briefly, about 20 mg of fat from the tissue samples were accurately weighed into test tubes. Then 2 mL sodium methoxide (Sigma-Aldrich Inc., Oakville, ON) was added, capped and vortexed for 1 min and incubated in a 50°C oven for 10 min. After incubation, 1 mL boron triflouride (14% in methanol, w:v, Sigma-Aldrich Inc., Oakville, ON) was added, capped and vortexed for 1 min and again incubated in a 50°C oven for an additional 10 min. After removal from the oven, 5 mL de-ionized water was added and vortexed followed by 5 mL hexane and vortexed. When the layers separated, an aliquot of the top layer was removed and transferred into a 2 mL gas chromatographic vial, capped and stored at -40°C until analyzed. Nonadecanoic acid methyl ester (C19:0; Nu-Chek Prep, Inc., MN, USA) was added to the fat samples as an internal standard.

Fatty acid methyl esters were quantified using a gas chromatograph (Hewlett Packard GC System 5890; Mississauga, ON) equipped with a flame ionization detector and SP-2560 fused silica capillary column (100 m with 0.2-μm film thickness; Supelco Inc., Oakville, ON). Samples were loaded on to the column via 1 μL splitless injections [37]. The initial oven temperature (120°C) was held for 15 min and then increased at 5°C/min to 160°C, and held for 15 min. Next the temperature was increased at 4°C/min to 240°C and held for 30 min. Inlet and detector temperatures were maintained at 220°C, and 275°C, respectively. Helium carrier gas flow rate through the column was 1.7 mL/min. Hydrogen flow to the detector was 34 mL/min, air flow rate was 320 mL/min and helium make-up gas flow rate was 29 mL/min. Peaks in the chromatograms were identified and quantified using pure methyl ester standards (Sigma-Aldrich Inc., Oakville, ON).

### Diet and blood component (glucose, cholesterol and triglycerides) analyses

Dietary Nitrogen was determined using the automated combustion technique ([38]; Carlo Erba^®^, Milan, Italy) and crude protein was calculated as the product of the Nitrogen value and 6.25, while fat content of the diets was determined by extraction of dried samples with di-ethyl ether for a minimum of six hours in a Goldfisch apparatus [39]. The gross energy in diets was determined by calorimetry ([17]; LECO AC-300, Leco Corporation, Michigan, USA). Blood glucose, cholesterol and triglycerides were analyzed on a VetTest 8008, Chemistry Analyzer (IDEXX Laboratories, Inc., Westbrook, Maine, USA) by using the slides for GLU (Code 2376), CHOL (Code 2358) and TRIG (Code 2396) (IDEXX Laboratories, Inc., Westbrook, Maine, USA), respectively after calibrating the equipment with appropriate standards.

### Statistical analysis

The data were analyzed for variance as a completely randomized design experiment, with four dietary treatments and n = 14 for each treatment for the growth data and with n = 7 for the fatty acid analyses. The analysis of variance was conducted using the PROC MIXED procedure of SAS (Statistical Analyses Systems Institute, Cary, NC) for the rat body weight, growth rate, feed consumption, the blood components (glucose, cholesterol and triglycerides), organ, muscle and adipose tissue (retroperitoneal and inguinal) weights and fatty acid composition of adipose tissues. Initial body weight and number experimental days were used as covariates for the growth and feed consumption data. The fixed effect and the differences among treatments were considered significant at probability values of p ≤ 0.05.

## Results

### Diet composition and growth performance of rats

The fat and protein content and gross energy of the diets were comparable (Table 1), because diets were prepared to be isonitrogenous and isocaloric, although the source of protein and energy varied among the diets. The concentration ranking, from high to low, in the experimental diets for both CLA isomers was SCLA, CLAM, CONM and CON (Table 2).

**Table 2:** Fatty acid (FA) composition of lipids in diets

	Treatment diets			
	
Fatty acids* (mg/100 mg)	CON	SCLA	CONM	CLAM
C14:0	0.06	0.09	5.26	5.47
C14:1 c	0.00	0.00	0.84	0.69
C16:0	5.09	5.44	24.32	23.06
C16:1 c	0.04	0.04	3.57	2.76
C18:0	3.03	3.05	15.92	19.21
C18:1 t11	0.01	0.01	0.19^†^	5.55
C18:1 c9	11.16	11.54	37.85	30.68
C18:2 c9, c12	77.74	69.20	5.56	8.34
C18:3	1.62	1.42	0.10	0.11
CLA c9, t11	0.03	4.64	0.29	0.49
CLA t10, c12	0.00	3.20	0.02	0.04
C20:4	0.02	0.03	0.07	0.08
UNS/Total FA	0.91	0.91	0.53	0.51
UNS/SAT	10.75	10.21	1.14	1.05
C18:2/C18:3	48.04	54.43	58.61	85.96

*denoted as Carbon chain length: number of unsaturated bonds in trans (t) or cis (c) position and location

^† ^Calculated from value for control beef in (Shah et al., 2006)

UNS – unsaturated FA, SAT – Saturated FA

CON – Control diet, SCLA – diet with 1.69% synthetic CLA replacing equivalent amount of the soybean oil, CONM – Diet containing control beef (see table 1), CLAM – diet containing high CLA beef (see table 1).

The meat (beef) diets (CONM and CLAM) contained four to five fold more saturated (C16:0 and C18:0) and monounsaturated fatty acids (MUFA) (C18:1 c9) but substantially less PUFA (C18:2 and C18:3) relative to the CON and SCLA diets, and were reflective of the differences in fatty acid composition between soybean oil and beef fat, which were the principal sources of fat in the diets. Vaccenic acid (C18:1 t11) concentration of the CLAM diet, which contained the high CLA beef was (5.5% of fatty acids) higher than of the CON, SCLA diets (0.01% of fatty acids) or the CONM diet, which contained the control beef (0.19% of fatty acids).

Body weights, intakes and growth rates have been reported for the first six weeks and for the duration of the feeding trial (Table 3). Although rat body weight differences at the start of the experiment were not evident, by six weeks the rats fed the SCLA diet were lighter (p = 0.02) than those fed the other diets, but this significance did not persist until the end of the study, which was nine weeks on average, but rats fed the SCLA diet tended (p = 0.07) to have a lower body weight. Growth rates followed the same pattern as the body weights, which may be related to lower total feed consumed by rats fed the CON or SCLA diets relative to those fed the CONM or CLAM diets. The over all protein consumed (product of feed consumed and diet-protein content/100) was higher for rats fed the meat containing diets (CONM or CLAM) than either the CON or the SCLA diet. The energy consumed (product of the feed consumed and the energy value of the diets) was lower for the CON and SCLA diets relative to that of the CONM and CLAM diets. These differences affected the energy requirement per gram of body weight gain. Thus efficiency of energy utilization for weight gain was better (lower energy required for each gram of gain) in rats fed the CON and SCLA diets than of those fed the CONM and CLAM diets. It was clear that animals consumed more of the meat containing diets relative to the casein containing diets.

**Table 3:** Effects of synthetic CLA or CLA beef on rat growth performance

	Treatment diets			
	
	CON	SCLA	CONM	CLAM
Initial BW (g)	85.5 ± 2.2	85.6 ± 2.0	86.0 ± 2.3	86.3 ± 2.1
BW at 6 wks (g)	410.6 ± 7.6^B^	387.0 ± 7.6^A^	413.1 ± 8.0^B^	421.6 ± 9.6^B^
Final BW at 9 wks (g)	486.1 ± 14.7	470.2 ± 11.7	500.3 ± 13.3	509.5 ± 18.4
DWG: 1–42 d (g/d)	7.7 ± 0.2^B^	7.2 ± 0.2^A^	7.8 ± 0.2^B^	8.0 ± 0.2^B^
DWG: overall (g/d)	6.4 ± 0.1	6.2 ± 0.1	6.7 ± 0.2	6.8 ± 0.2
FI: 1–42 d (g)	1053 ± 46^A^	932 ± 34^A^	1333 ± 81^B^	1294 ± 53^B^
FI: overall (g)	1708 ± 74^A^	1566 ± 95^A^	2149 ± 159^B^	2173 ± 156^B^
Daily FI (g/d)	27.1 ± 1.1^A^	24.9 ± 1.1^A^	34.0 ± 2.5^B^	33.8 ± 1.9^B^
Protein intake (g)	333.2 ± 15.0^A^	314.8 ± 19.1^A^	412.9 ± 26.5^B^	458.5 ± 24.1^B^
GE intake (kcal)	7381 ± 331^A^	6608 ± 400^A^	9066 ± 583^B^	8887 ± 466^B^
GE/BWG: overall (kcal/g)	18.5 ± 0.85^A^	17.1 ± 0.64^A^	22.1 ± 1.05^B^	21.7 ± 0.94^B^
CLA c9, t11 consumed (g)	0.04	5.31	0.34	0.81
CLA t10, c12 consumed (g)	-	3.45	0.02	0.06

A, B: Means (± standard error) in a row denoted by a different letter differ (p < 0.05); n = 14

BW – Body weight, DWG – daily weight gain, FI – feed intake, GE – gross energy,

CON – Control diet, SCLA – diet with 1.69% synthetic CLA replacing and equivalent amount of soybean oil, CONM – Diet containing control beef (see table 1), CLAM – diet containing high CLA beef (see table 1).

Protein intake – product of diet protein content and FI overall/100.

GE intake – product of diet GE and FI overall.

Consumed CLA c9, t11 or CLA t10, c12 – calculated from means, CLA c9, t11 or CLA t10, c12 percent in diet × diet fat content × FI overall/10000; respectively.

### Carcass, organ and muscle weights

The carcass weights, similar to the final body weights of the rats were not affected by the different diets and only rats fed the SCLA diet tended (p = 0.11) to have a lower carcass weight (Table 4). Differences due to diet were not observed for organ weights, but when expressed as a percent of body weight, rats fed the CONM diet had lower percentages for heart, liver and kidney than for rats fed the SCLA diet. Organ weight as percent of body weight in rats fed the CON or CLAM diets did not differ, except for liver weight as percent of body weight, where this value was greater in the rats fed the CON and SCLA diets than those observed for the rats receiving the CONM diet. The diets did not affect the weights of the soleus and gastrocnemius muscles or the percentage of the muscle weights to body weight.

**Table 4:** Carcass components, cellularity of fat pads and concentrations of blood glucose, cholesterol and triglycerides in the rats

	Treatment diets			
	
	CON	SCLA	CONM	CLAM
Carcass weight (g)	395.33 ± 11	374.73 ± 10	392.90 ± 10	402.86 ± 13
Heart (g)	1.50 ± 0.03	1.51 ± 0.04	1.44 ± 0.04	1.53 ± 0.04
Heart/BW (%)	0.31 ± 0.01^AB^	0.32 ± 0.01^B^	0.29 ± 0.01^A^	0.30 ± 0.01^AB^
Liver (g)	13.74 ± 0.31	13.42 ± 0.29	13.18 ± 0.53	14.06 ± 0.53
Liver/BW (%)	2.85 ± 0.08^B^	2.86 ± 0.06^B^	2.64 ± 0.09^A^	2.77 ± 0.07^AB^
Kidney (g)	3.13 ± 0.08	3.22 ± 0.08	3.11 ± 0.06	3.17 ± 0.06
Kidney/BW (%)	0.65 ± 0.02^AB^	0.69 ± 0.02^B^	0.62 ± 0.01^A^	0.63 ± 0.02^A^
Soleus (g)	0.33 ± 0.10	0.20 ± 0.01	0.22 ± 0.01	0.21 ± 0.01
Soleus/BW (%)	0.06 ± 0.016	0.04 ± 0.002	0.04 ± 0.001	0.04 ± 0.002
Gastrocnemius (g)	2.73 ± 0.12	2.57 ± 0.09	2.72 ± 0.06	2.84 ± 0.06
Gastrocemius/BW (%)	0.57 ± 0.03	0.55 ± 0.02	0.55 ± 0.02	0.56 ± 0.02
**Retroperitoneal fat (RP)**				
Weight (g)	12.85 ± 1.26^B^	8.68 ± 1.07^A^	16.86 ± 1.58^C^	16.98 ± 1.91^C^
RP/BW (%)	2.61 ± 0.22^B^	1.80 ± 0.19^A^	3.32 ± 0.26^C^	3.22 ± 0.25^C^
n × 10^7 ^cells/g	26.90 ± 7.16	9.38 ± 2.10	22.28 ± 5.95	23.39 ± 4.44
**Inguinal fat (IG)**				
Weight (g)	10.12 ± 0.98^AB^	7.62 ± 0.48^A^	11.69 ± 0.86^B C^	12.80 ± 1.54^C^
IG/BW (%)	2.06 ± 0.18^B^	1.60 ± 0.07^A^	2.31 ± 0.13^C^	2.42 ± 0.21^C^
n × 10^7 ^cells/g	20.15 ± 4.18^AB^	26.01 ± 5.94^B^	28.88 ± 5.56^B^	7.26 ± 1.28^A^
**Blood components**				
Glucose (g/L)	1.27 ± 0.04 b^B^	1.17 ± 0.05^A^	1.39 ± 0.05^B^	1.37 ± 0.06^B^
Cholesterol (g/L)	0.60 ± 0.03	0.59 ± 0.05	0.59 ± 0.04	0.63 ± 0.07
Triglycerides (g/L)	0.73 ± 0.08	0.79 ± 0.06	0.77 ± 0.06	1.04 ± 0.13

A-C: Means (± standard error) in a row denoted by a different letter differ (p < 0.05); n = 14

BW – body weight

CON – Control diet, SCLA – diet with 1.69% synthetic CLA replacing an equivalent amount of soybean oil, CONM – Diet containing control beef (see table 1), CLAM – diet containing high CLA beef (see table 1).

### Adipose tissue weights and adipocyte number

**Figure 1:**
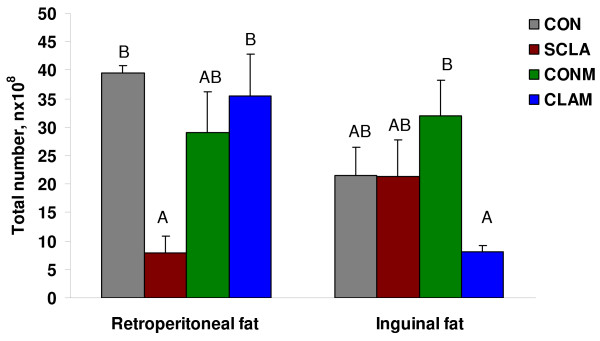
**Comparison of the total number of adipocytes in fat tissues from rats fed with various diets**. A, B: Means (± standard error) denoted by a different letter differ (p < 0.05); n = 14. CON – Control diet, SCLA – diet with 1.69% synthetic CLA replacing and equivalent amount of soybean oil, CONM – Diet containing control beef (from steers not provided sunflower seeds (SS) during their production, with composition; 97% dry matter (DM), 52.6% protein and 38.8% lipids in DM), CLAM – diet containing high CLA beef (from steers fed 14% SS during their production with composition; 95% dry matter (DM), 60.6% protein and 28.5% lipids in DM).

The RP fat pad weights were lowest (p < 0.01) in rats fed the SCLA diet at 8.68 g and lower than that of rats fed the CON diet (12.85 g), which was lower than of rats fed the meat containing diets (16.86 and 16.98 g for the rats fed CONM and CLAM diets, respectively), which were not different from each other. The weights for this tissue as a percent of body weight followed the same pattern as tissue weights. Because of large standard errors, differences in adipocyte density (n × 10^7^/g) of the retroperitoneal adipose tissue of rats fed the different diets were not observed. However, when the cell density was multiplied by the tissue weight to obtain total cells in tissue (n × 10^8^; Figure 1), the provision of either the CON or the CLAM diets led to similar and the highest (p = 0.04) cell numbers, which were greater than of rats fed the SCLA diet.

The weight of the IG tissue of rats fed the CLAM diet was higher (p < 0.01) than that of rats fed the CON, which was greater (p = 0.01) than that of rats fed the SCLA diet. Inguinal tissue weights as percent of body weight was greatest for rats fed either CONM or the CLAM diets, which were greater than of rats fed the CON diet and that of rats fed the SCLA was the least (p < 0.01). Adipocyte density was higher (p < 0.01) in rats fed the SCLA or the CONM diets and greater (p < 0.01) than in rats fed the CLAM diet as a result the total cells were lower (p = 0.02) in rats receiving the CLAM relative to those fed the CONM diet (Figure 1).

### Blood glucose, cholesterol and triglycerides

The fasting blood glucose concentration (Table 4) of rats fed the SCLA was lower (p < 0.01) than of rats fed meat containing diets and the CON diet. Differences in fasting cholesterol were not observed for the rats, despite substantial variation in protein source and fat type in the diets. However in rats fed the CLAM diet a trend (p = 0.07) towards higher triglycerides was observed relative to that in rats fed the other diets.

### Fat content and fatty acids composition of adipose tissues

Fat content of the RP adipose tissue was comparable for the rats fed the different diets and ranged between 80.15 to 87.68% (Table 5) and that of the IG tissue ranged between 76.94% for the rats fed the SCLA diet and 85.09% fat content of rats fed the CON diet (Table 6). The fat content of the IG tissue of rats fed the SCLA diet tended (p = 0.08) to be lower than of rats fed the CON diet while differences were not observed for rats fed the other diets.

**Table 5:** Effects of synthetic CLA or high CLA beef on fatty acid composition of retroperitoneal fat of the rats

	Treatment diets			
	
	CON	SCLA	CONM	CLAM
Lipid (% tissue)	81.33 ± 2.32	80.15 ± 3.80	84.54 ± 2.43	87.68 ± 5.24
Fatty acid concentration (mg/100 mg of detected fatty acids)				
C14:0*	1.13 ± 0.096	1.06 ± 0.065	1.67 ± 0.089	1.99 ± 0.103
C14:1 c	0.01 ± 0.004^A^	0.01 ± 0.005^A^	0.46 ± 0.016^B^	0.44 ± 0.015^B^
C16:0	22.92 ± 0.83	25.37 ± 0.51	23.88 ± 0.48	25.28 ± 0.71
C16:1 c	4.79 ± 0.22^B^	2.88 ± 0.14^A^	6.78 ± 0.28^C^	6.31 ± 0.32^C^
C18:0	3.74 ± 0.13^A^	3.47 ± 0.30^A^	4.63 ± 0.33^B^	6.00 ± 0.26^C^
C18:1 t11	0.00 ± 0.73	0.65 ± 0.63	1.01 ± 0.63	2.14 ± 0.83
C18:1 c9	28.86 ± 0.66^A^	25.10 ± 0.77^A^	52.48 ± 0.91^B^	48.31 ± 1.54^B^
C18:2 c9, c12	30.49 ± 2.46^C^	26.60 ± 1.40^B^	2.58 ± 0.36^A^	3.50 ± 0.39^A^
C18:3	3.0 ± 0.07^C^	2.33 ± 0.03^B^	0.17 ± 0.01^A^	0.14 ± 0.01^A^
CLA c9, t11	0.01 ± 0.002^A^	4.50 ± 0.408^C^	0.38 ± 0.011^B^	0.60 ± 0.041^B^
CLA t10, c12	0.10 ± 0.003^A^	3.16 ± 0.081^B^	0.23 ± 0.006^A^	0.19 ± 0.007^A^
C20:4	0.41 ± 0.17	0.21 ± 0.014	0.28 ± 0.003	0.21 ± 0.010
C22:5	0.18 ± 0.007	0.39 ± 0.004	0.49 ± 0.000	0.42 ± 0.000
C22:6	0.78 ± 0.011	0.60 ± 0.007	0.81 ± 0.000	0.86 ± 0.000
UNS/Total FA	0.72 ± 0.02^B^	0.69 ± 0.01^AB^	0.68 ± 0.01^AB^	0.65 ± 0.01^A^
UNS/SAT	2.60 ± 0.19^B^	2.27 ± 0.16^AB^	2.18 ± 0.06^AB^	1.91 ± 0.09^A^
C18:2/C18:3	9.99 ± 0.58^A^	14.43 ± 0.27^AB^	20.36 ± 3.15^BC^	25.66 ± 2.42^C^

A-C: Means (± standard error) in a row denoted by a different letter differ (p < 0.05); n = 7

*denoted as Carbon chain length: number of unsaturated bonds in trans (t) or cis (c) position and location

CON – Control diet, SCLA – diet with 1.69% synthetic CLA replacing an equivalent amount of soybean oil, CONM – Diet containing control beef (see table 1), CLAM – diet containing high CLA beef (see table 1).

The fatty acid profiles of the two fad pads were comparable and influenced by the fatty acid composition of the diets provided to the rats. Although the total profiles are reported in the tables, only relevant components will be highlighted in this section. In the RP adipose tissue the major saturated fatty acid was C16:0, which is indicative of *de novo *synthesis in the animals fed the CON and SCLA diets and accounted for a quarter of the total fatty acids detected and differences due to diet were not evident. The range for C18:0 was from 3.47 to 6.00% and the values were higher (p < 0.01) in rats fed the CLAM diets relative to the others, which is suggestive of the extent of biohydrogenation of the oil from the SS fed to the beef steers to produce the high CLA beef. A greater amount of the high CLA beef was incorporated in the CLAM relative to the control beef in the CONM diet, due to the difference in fat content of the two meats, further elevating the supply of saturated fatty acids in the diet. For the same reason the total unsaturated fatty acids and their ratio to the saturated fatty acids were least in the RP tissues of rats fed the CLAM diet relative to those fed the CON diet and were reflective of the fatty acid composition of the fat source employed in the preparation of the diets. The C18:1 c9, C18:2 c9, 12 and C18:3 c9, 12, 15 concentrations in the RP fat were reflective of the principal fat source in the diets and C18:1 concentrations were higher in animals fed the control beef (CONM) while the diets prepared with soybean oil had higher levels of the C18:2 and C18:3. Although the diets did not contain the elongated ω-3 fatty acids (C22:5 and C22:6) these were detected in the RP fat suggesting significant endogenous synthesis of these fatty acids. Both CLA isomers were found in this tissue, and were affected by dietary treatment, thus rats fed the SCLA diet had substantially higher (p < 0.01) concentrations of these fatty acids relative to rats fed the other diets. The CLA c9 t11 was higher in rats fed the beef containing diets relative to those receiving the CON diet, but the rats fed the CLAM diet had only numerically higher amounts in the retroperitoneal tissue relative to those fed the CONM diet. Although not significant, the concentration of C18:1 t11 was twice as high in the tissues of rats fed the CLAM diet relative to those fed the CONM diet.

The fatty acid composition of the IG fat pads (Table 6) was similar to that of RP pads. However, unlike observations in the RP fat pads, the C14:0 tended (p = 0.11) to be elevated in rats fed the beef containing diets (CONM and CLAM) relative to the concentrations of this fatty acid in those fed the CON diet. Among the other saturated fatty acids, C16:0 comprised about 25% of the total fatty acids. The concentrations of the C18:0 were highest in rats fed the CLAM diet. In the IG adipose tissue the ratios of unsaturated fatty acids to total fatty acids, or to saturated fatty acids were higher (p = 0.02, < 0.01) in the rats fed the CON diet relative to those fed the CLAM diet. The concentrations of C18:1 c9; C18:2 c9, 12; and C18:3 c9, 12, 15 were as observed for the RP fat pad. The CLA isomers were highest in rats fed the SCLA diet, as observed in the RP tissue, and rats fed the CLAM diet demonstrated only numerical increases in concentrations of the CLA c9, t11 and CLA t10, c12. Although only a trend (p = 0.14), rats fed the CLAM diet had five times more C18:1 t11 than rats fed the CONM diet.

**Table 6:** Effects of synthetic CLA or high CLA beef on fatty acid composition of inguinal fat of the rats

	Treatment diets			
	
	CON	SCLA	CONM	CLAM
Lipid (% tissue)	85.09 ± 2.2	76.94 ± 1.68	81.30 ± 2.50	84.22 ± 4.42
Fatty acid concentration (mg/100 mg of detected fatty acids)				
C14:0*	0.58 ± 0.094	1.48 ± 0.441	2.03 ± 0.097	2.48 ± 0.39
C14:1 c	0.00 ± 0.005^A^	0.00 ± 0.005^A^	0.55 ± 0.013^B^	0.50 ± 0.42^B^
C16:0	22.97 ± 1.59	25.45 ± 1.08	23.50 ± 0.29	24.30 ± 2.25
C16:1 c	5.14 ± 0.23^AB^	3.53 ± 0.16^A^	8.05 ± 0.29^C^	7.56 ± 0.66^BC^
C18:0	3.51 ± 0.29^A^	3.02 ± 0.07^A^	4.15 ± 0.40^AB^	5.04 ± 0.27^B^
C18:1 t11	0.42 ± 0.91	0.64 ± 1.72	0.71 ± 1.17	3.67 ± 1.21
C18:1 c9	27.56 ± 2.32^A^	24.53 ± 2.01^A^	52.23 ± 0.73^C^	46.44 ± 4.10^B^
C18:2 c9, c12	30.77 ± 2.28^B^	26.63 ± 1.77^B^	2.87 ± 0.19^A^	3.50 ± 0.99^A^
C18:3	3.25 ± 0.06^C^	2.58 ± 0.05^B^	0.18 ± 0.01^A^	0.29 ± 0.02^A^
CLA c9, t11	0.00 ± 0.04^A^	4.54 ± 0.08^C^	0.42 ± 0.01^B^	0.79 ± 0.09^B^
CLA t10, c12	0.19 ± 0.002^A^	2.92 ± 0.031^B^	0.08 ± 0.002^A^	0.32 ± 0.008^A^
C20:4	0.53 ± 0.026^B^	0.27 ± 0.024^A^	0.09 ± 0.015^A^	0.05 ± 0.012^A^
C22:5	0.42 ± 0.005	0.25 ± 0.006	0.16 ± 0.003	0.50 ± 0.001
C22:6	0.63 ± 0.015	0.49 ± 0.009	0.69 ± 0.009	0.80 ± 0.003
UNS/Total FA	0.72 ± 0.01^B^	0.69 ± 0.01^AB^	0.69 ± 0.00^AB^	0.67 ± 0.01^A^
UNS/SAT	2.61 ± 0.1^B^	2.24 ± 0.1^AB^	2.22 ± 0.04^AB^	2.05 ± 0.1^A^
C18:2/C18:3	9.18 ± 0.32^A^	12.94 ± 0.21^AB^	15.98 ± 2.46^B^	16.66 ± 4.15^B^

A-C: Means (± standard error) in a row denoted by a different letter differ (p < 0.05); n = 7

*denoted as Carbon chain length: number of unsaturated bonds in trans (t) or cis (c) position and location

CON – Control diet, SCLA – diet with 1.69% synthetic CLA replacing an equivalent amount of soybean oil, CONM – Diet containing control beef (see table 1), CLAM – diet containing high CLA beef (see table 1).

The ratios of C18:2 to C18:3 were higher in the diets than in the two adipose tissues that were studied and may be largely due to the presence of elongated ω-3 fatty acids in the tissues but not in the diets. In the diets, the ratios ranged between 48.04 and 85.96 for the CON and CLAM diets respectively, but this ratio ranged between 9.99 and 25.66 in the RP fat pad and 9.18 and 16.66 in the IG fat pads for rats fed the same diets and differed significantly.

## Discussion

Among the objectives of the study was the establishment of the effects of the diet containing beef with the bio-formed CLA under conditions where the diets were balanced for protein and fat content, but the nature of the proteins and the fatty acid composition of the fat varied among the diets on adipose tissue characteristics. The inclusion rate of the synthetic CLA was high and comparable to that in the previous study and represented the recommended level of dietary incorporation for CLA. The increase in content of the two CLA isomers in the diets containing the high CLA beef was comparable to concentrations achieved previously [17], even though it was necessary to add casein in the CLAM and CONM diets to achieve the 20% protein without affecting the fat content of the diets.

The feed consumption of the CON and SCLA was less than of CONM or CLAM diets and the results are in concurrence with previous reports [40] which indicate poor consumption rates for low protein or non-appetizing diets, indicating a strong preference for meat containing diets.

Although carcass weights were greater for rats in the present study for nine week old rats than those reported for 11 wk old rats, the liver weights were lower than reported weights [16] and may be related to age of the rats at euthanasia or differences between strains of rats (Sprague-Dawley vs Wistar). Yet, among the same strain of rats, the liver weights were lower in the present study, in spite of the rats being older than those in the previous study [17]. It may be indicative of attainment of mature size, after which a further increase in organ weight may not occur, even though body weight continues to increase in response to feed and age. The weight increase response may register in weight of adipose tissue.

The RP adipose tissue weights observed in the present study were greater than those reported [16], even though the rats were older in their study and attributable to being a characteristic of Sprague-Dawley rats as compared to Wistar rats. However, relative to the previous report [17] for Wistar rats, the weights of this tissue in this study were higher, which could be an age of rat related factor (six vs nine weeks). The synthetic CLA has always been known to reduce the weight and cell number of the RP fat pads [17] and the effect on this tissue in the present study was consistent with previous observations.

Inguinal adipose tissue weights were higher than those reported [16,17] but similar to those reported for IG tissue weights of rats fed high fat diets [34], probably age and strain of rats may be operative for the variations observed.

The relationship among diet components, tissue cell number and tissue weight is unclear. Mir and co-workers [17] observed that cell density and total cell number in the two adipose tissues were markedly greater in rats fed sunflower oil in comparison to those fed soybean oil, yet tissue weights were similar, and even though synthetic CLA provision decreased RP tissue weights, total cell number were not different from those of rats fed the control diet. Furthermore, synthetic CLA failed to affect the IG adipose tissue weight or cell density when compared to the control diet in the previous study [17] and similarly, IG tissue cell number were comparable for rats fed CON and SCLA diets in the present study. However, when rats were fed the CLAM diet the tissue weights were comparable to those of rats fed the CONM but the cell density and total number were markedly lower (p < 0.01) and consistent with previous observations for the IG tissue [17]. It is of importance to note that the total CLA c9, t11 consumed over the duration of the trial by rats fed the CLAM diet was 0.81 g and the highest between rats fed the beef containing diets. Also the dietary C18:1 t11 was highest in this diet; and it is known that this fatty acid contributes to endogenous synthesis of CLA c9, t11 [41]. The CLA c9, t11 is an anti-proliferative, thus its anticarcinogenic effect, and *in vitro*, it appears to inhibit cell proliferation of preadipocytes [6,7], but encourages fat accumulation in the remaining cells, which may explain the observed effect on cell density and IG tissue weight of rats fed the CLAM diet. Previously Chardigny and co workers [42] have reported that in some strains of rats the size of the IG tissues was reduced by dietary synthetic CLA while livers became fat infiltrated. Although an effect on the liver was not observed in the present study or in the previous study [17], it appears that the bio-formed CLA provided as beef affected cell numbers of this tissue.

The amounts of bio-formed CLA c9, t11 consumed by the rats through the duration of the study from the CLAM diet was approximately 15% of the synthetic CLA c9, t11 in rats fed the SCLA diet, thus, IG fat pad weight was not affected, but the cell numbers were diminished. The effect on the decreased cell number is consistent with a previous observation [17], where the dietary protein was 12%. It has been observed that the percent reduction in fat mass of human subjects consuming a CLA triglyceride was a consistent 8% over a 24-month period relative to only a 5% decline in subjects consuming the CLA free acid [19], thus it can be hypothesized that the marginal greater effect of the CLA triglyceride could be due to the effect of the CLA at the Sn-2 position. Digestion, absorption and probably utilization of fatty acids in the Sn-2 position are reported to progresses differently from those in the Sn-1 and Sn-3 position [43]. Chardigny and co-workers [44] reported that rumenic acid in the Sn-2 position was found to a greater extent in the carcass, while that from the Sn-1 or 3 positions led to increased yield of energy (recovered in exhaled carbon dioxide), even though differences in digestibility of the rumenic acid provided as free fatty acid or as triacylglycerol did not exist [45]. In earlier studies it has been reported that the CLA in muscle fat is largely located at the Sn-2 position [46] unlike that in milk fat [44]. The location of this fatty acid in the triacylglycerol in beef may confer increased efficacy and explain the heightened responses of cancer protection among milk consumers referred to by Knekt and co-workers [29] and observed by Hubbard and co-workers [30]. The synthetic and bio-formed CLA would be absorbed as free fatty acid or as Sn2 mono-acylglycerol, respectively and may bind to different receptors in RP and IG tissue leading to the differential response observed in the tissues to the two forms of CLA. Although similar response has been observed both in this study and in the previous study [17] the cause for such a response needs definition.

Transfer of dietary fatty acids to the adipose tissues was observed in the rats. Greater levels of the C18:1c9 and C18:0 were observed in rats fed the meat containing diets with further elevated levels of C18:0 in the rats fed the CLAM diet indicating the elevated biohydrogenation of the linoleic acid in the dietary SS provided to the steers during production of the beef. Since soybean oil was added to the CON and SCLA diets, greater proportions of C18:2 and C18:3 were present in both the adipose tissues than in rats fed the meat containing diets, as has been observed in pigs [47]. The provision of C18:2 and C18:3 is known to suppress steroyl regulatory element binding protein -1 leading to reductions in fatty acid synthase gene transcription [48] and may be associated with the observation of diminished weight of the RP adipose tissue in rats fed the CON diet in the present study relative to those fed the meat containing diets.

It is of interest to mention that even though the casein diets contained soybean oil, a high-PUFA oil relative to beef fat, a difference in the fasting plasma cholesterol was not observed suggesting that the effect of beef on plasma cholesterol profiles needs to be revisited. There may be advantage to using animal models which respond to dietary alterations through changes in plasma cholesterol concentration as in humans such as guinea pigs [49] to assess the value of high CLA beef in controlling plasma cholesterol and the lipoprotein class in which the cholesterol occurs. Development of knowledge regarding the distribution of the cholesterol among the various lipoproteins as influenced by high CLA beef would provide the necessary information to promote the benefits of consumption of beef. Despite substantially higher values for adipose tissues weights as percent of body weight, fasting plasma glucose concentrations were similar for rats fed the CON, CONM and CLAM diets, suggesting that the adipose tissues were equally effective in removing glucose out of circulation. The low plasma glucose for the rats fed SCLA may be related to the specific effects of synthetic CLA on the RP adipose tissue and the diminished intake of the diet relative to the beef containing diets, leading to lower glucose load and improved management. The trend towards the increased plasma triacylglycerol in CLAM fed rats may be suggestive of lipid saturation of the adipocytes in either tissue.

## Conclusion

Despite the substantially lower concentrations of the CLA consumed from the high-CLA beef diets, relative to that by the rats fed synthetic CLA, a decrease in cellularity of the IG fat depot was observed. The effects observed previously [17] and the current study, indicate that the bio-formed CLA affects mainly IG fat pads at substantially lower concentrations than synthetic CLA free acid, which appears to decrease the size and total number of cells in the RP adipose tissue. Although more studies on the effect of CLA are required in the future, the present study suggests that dietary CLA from high CLA beef as well as synthetic CLA may influence lipid storage potential of the adipose tissues through affecting tissue size or cellularity and may not detrimentally affect plasma cholesterol concentrations.

## Abbreviations

CLA: conjugated linoleic acid; CON: Control diet; CLAM: CLA beef diet; CONM: control meat (beef) diet; c: cis; IG: inguinal; MUFA: Monounsaturated fatty acids; PUFA: polyunsaturated fatty acids; RP: Retroperitoneal; SCLA: synthetic CLA diet; t: trans.

## Competing interests

The authors declare that they have no competing interests.

## Authors' contributions

MLH was a Research Associate who conducted a significant portion of the fatty acid analysis and compiled the first draft of the manuscript. EKO, as over all co-ordinator of the CLA Network project that was funded by the Alberta Agriculture Research Institute and HN was the Co-op student who conducted the study under the supervision of PSM who was the project leader for the Beef Module of the CLA-Network project and is the corresponding author of this manuscript.

## References

[B1] Ip C, Singh M, Thompson HJ, Scimeca JA (1994). Conjugated linoleic acid suppresses mammary carcinogenesis and proliferative activity of the mammary gland in the rat. Cancer Res.

[B2] Parodi PW (1997). Cow's milk fat components as potential anticarcinogenesis agents. J Nutr.

[B3] Kimoto N, Hirose M, Futakuchi M, Iwata T, Kasai M, Shirai T (2001). Site-dependent modulating effects of conjugated linoleic acids from safflower oil in a rat two-stage carcinogenesis model in female Sprague-Dawley rats. Cancer Lett.

[B4] Lee KN, Kritchevsky D, Pariza MW (1994). Conjugated linoleic acid and atherosclerosis in rabbits. Atherosclerosis.

[B5] Nicolosi RJ, Rogers EJ, Kritchevsky D, Scimeca JA, Huth PJ (1997). Dietary conjugated linoleic acid reduces plasma lipoproteins and early atherosclerosis in hypercholesterolemic hamsters. Artery.

[B6] Brodie AE, Manning VA, Ferguson KR, Jewell DE, Hu CY (1999). Conjugated linoleic acid inhibits differentiation of pre- and post-confluenct 3T3-L1 preadipocytes but inhibits cell proliferation only in preconfluent cells. J Nutr.

[B7] Satory DL, Smith SB (1999). Conjugated linoleic acid inhibits proliferation but stimulates lipid filling of murine 3T3-L1 preadipocytes. J Nutr.

[B8] Brown JM, Boysen MS, Jenson SS, Morrison RF, Storkson J, Lea-Currie E, Pariza MV, Mandrup S, McIntosh MK (2003). Isomer-specific regulation of metabolism and PPARγ signaling by CLA in human preadipocytes. J Lipid Res.

[B9] Brown JM, Boysen MS, Chung S, Fabiyi O, Morrison RF, Mandrup S, McItosh MK (2004). Conjugated linoleic acid induces human adipocyte delipidation. Autocrine/paracrine regulation of MEK/ERK signaling by adipocytokines. J Biol Chem.

[B10] Kang K, Liu W, Albright KJ, Park Y, Pariza MW (2003). Trans-10, cis-12 CLA inhibits differentiation of 3T3-L1 adipocytes and decreases PPARγ expression. Biochem Biophys Res Commun.

[B11] Granlund L, Pedersen JI, Nebb HI (2005). Impaired lipid accumulation by trans 10, cis 12 CLA during adipocyte differentiation is dependent on timing and length of treatment. Biochim Biophys Acta.

[B12] He ML, Hnin TM, Kuwayama H, Mir PS, Okine EK, Hidari H (2006). Effect of Various Conjugated Linoleic Acids or Linoleic Acid on Glycerol-3-Phosphate Dehydrogenase Activity and Fatty Acids Accumulation in Differentiating 3T3-L1 Adipocytes: Interaction of Isomer Type, Treatment Period and Dose. Lipids.

[B13] Park Y, Albright KJ, Liu W, Storkson JM, Cook ME, Pariza MW (1997). Effect of conjugated linoleic acid on body composition in mice. Lipids.

[B14] Dugan MER, Aalhus JL, Schaefer AL, Kramer JKG (1997). The effect of conjugated linoleic acid on fat to lean repartitioning and feed conversion in pigs. Can J Anim Sci.

[B15] Szymczyk B, Pisulewski P, Szczurek W, Hanczakowski P (2000). The effect of feeding conjugated linoleic acid (CLA) on rat growth performance, serum lipoproteins and subsequent lipid composition of selected rat tissues. J Sci Food Agric.

[B16] Poulos SP, Sisk M, Hausman DB, Azain MJ, Hausman GJ (2001). Pre- and Postnatal dietary conjugated linoleic acid alters adipose development, body weight gain and body composition in Sprague-Dawley rats. J Nutr.

[B17] Mir PS, Okine EK, Goonewardene L, He ML, Mir Z (2003). Effects of synthetic conjugated linoleic acid (CLA) or bio-formed CLA as high CLA beef on rat growth and adipose tissue development. Can J Anim Sci.

[B18] Riserus U, Berglund L, Vessby B (2001). Conjugated linoleic acid (CLA) reduced abdominal adipose tissue in obese middle-aged men with signs of the metabolic syndrome: a randomized controlled trial. Int J Obesity.

[B19] Gaullier J-M, Halse J, Hoye K, Kristiansen K, Fagertun H, Vik H, Gudmundsen O (2005). Supplementation with conjugated linoleic acid for 24 months is well tolerated by and reduces body fat mass in healthy, overweight humans. J Nutr.

[B20] Bauman DE, Barbano DM, Dwyer DA, Griinari JM (2000). Technical note: Production of butter with enhanced conjugated linoleic acid for use in biomedical studies with animal models. J Dairy Sci.

[B21] Kepler C, Hirons KP, McNeill JJ, Tove SB (1966). Intermediates and products of the biohydrogenation of linoleic acid by Butyrovibrio fibrisolvens. J Biol Chem.

[B22] Griinari JM, Cori BA, Lacy SH, Chouinard PY, Nurmela KVV, Bauman DE (2000). Conjugated linoleic acid is synthesized endogenously in lactating dairy cows by Delta (9) – desaturase. J Nutr.

[B23] Kucuk O, Hess BW, Ludden PA, Rule DC (2001). Effect of forage: concentrate ratio on ruminal digestion and duodenal flow of fatty acids in ewes. J Anim Sci.

[B24] Beaulieu AD, Drackley JK, Merchen NR (2002). Concentrations of conjugated linoleic acid (cis-9, trans-11-octadecadienoic acid) are not increased in tissue lipids of cattle fed a high-concentrate diet supplemented with soybean oil. J Anim Sci.

[B25] Shantha NC, Crum AD, Decker EA (1994). Evaluation of conjugated linoleic acid concentrations in cooked beef. J Agric Food Chem.

[B26] Mir PS, Ivan M, He ML, Pink BM, Okine E, Goonewardene L, McAllister TA, Weselake RJ, Mir Z (2003). Dietary manipulation to increase conjugated linoleic acids and other desirable fatty acids in beef: A review. Can J Anim Sci.

[B27] Mir PS, Mir Z, Kuber PS, Gaskins CT, Martin EL, Dodson MV, Elias Calles JA, Johnson KA, Busboom JR, Wood AJ, Pittengers GJ, Reeves JJ (2002). Growth, carcass characteristics, muscle conjugated linoleic acid (CLA) content and plasma insulin concentrations in response to intravenous glucose challenge in high percentage Wagyu, Wagyu × Limousin and Limousin steers fed sunflower oil containing diets. J Anim Sci.

[B28] Shah MA, Mir PS, Aalhus JL, Basarab J, Okine EK (2006). Effects of sunflower oil seed inclusion in finishing diets for steers on performance, carcass characteristics, muscle and adipose fatty acid composition and meat quality. Can J Anim Sci.

[B29] Knekt P, Jarvinen R, Seppanen R, Pukkala E, Aroma A (1996). Intake of dairy products and the risk of breast cancer. Brit J Cancer.

[B30] Hubbard NE, Lim D, Erickson KL (2006). Beef tallow increases the potency of conjugated linoleic acid in the reduction of mouse mammary tumor metastasis. J Nutr.

[B31] Torre ADL, Debiton E, Juanéda P, Durand D, Chardigny J-M, Barthomeuf C, Bauchart D, Gruffat D (2006). Beef conjugated linoleic acid isomers reduce human cancer growth even when associated with other beef fatty acids. Brit J Nutr.

[B32] Olfert ED, Cross BM, McWilliam AA, Canadian Council on Animal Care (1993). Guide to the care and use of experimental animals.

[B33] Cartwright AL, Hausman, Martin (1987). Determination of adipose tissue cellularity. Biology of the Adipocyte.

[B34] Marques BG, Hausman DB, Latimer AM, Kras KM, Grossman BM, Martin RJ (2000). Insulin-like growth factor 1 mediates high-fat diet induced adipogenesis in Osborne Mendel rats. Am J Physiol Regul Integr Comp Physiol.

[B35] Jiang J, Bjoerck L, Fonden R, Emanuelson M (1996). Occurrence of conjugated cis-9, trans 11-octadecadonic acid in bovine milk: effects of feed and dietary regimen. J Dairy Sci.

[B36] Lock AL, Garnsworthy PJ (2002). Independent effects of dietary linoleic and linolenic fatty acids on the conjugated linoleic acid content of cows' milk. Anim Sci.

[B37] Kramer JKG, Fellner V, Dugan MER, Sauer FD, Mossoba MM, Yurawecz MP (1997). Evaluating acid and base catalysts in the methylation of milk and rumen fatty acids with special emphasis on conjugated dienes and total trans fatty acids. Lipids.

[B38] Smith KA, Tabatabai MA, Smith KA, Cresser MS (2004). Automated instruments for the determination of total carbon, hydrogen, nitrogen, sulphur and oxygen. Soil and environmental analysis modern instrumental techniques.

[B39] Cunniff PA, AOAC (1984). Official Methods of Analysis of the Association of Official Analytical Chemists. Method 96806.

[B40] Cherala G, Shapiro BH, D'mello AP (2006). Two low protein diets differentially affect food consumption and reproductive performance in pregnant and lactating rats and long-term growth in their offspring. J Nutr.

[B41] Kuhnt K, Kraft J, Moeckel P, Jahreis G (2006). Trans-11-18:1 is effectively Δ9-desaturated compared with trans-12-18:1 in humans. Brit J Nutr.

[B42] Chardigny JM, Arnal MA, Juanéda P, Genty M, Grégoire S, Sebédio JL (2001). Effect of cis-9, trans-11 and 10trans, 12cis-CLA isomers in two strains of mice. Ann Nutr Metab.

[B43] Berry SEE, Sanders TAB (2005). Influence of triacylglycerol structure of stearic acid-rich fats on post prandial lipaemia. Proc Nutr Soc.

[B44] Chardigny JM, Masson E, Sergiel JP, Darbois M, loreau O, Noel JP, Sebedio J-L (2003). The position of rumenic acid on triacylglycerols alters its bioavailability in rats. J Nutr.

[B45] Plourde M, Sergiel JP, Chardigny JM, gregoire S, Angers P, Sebedio JL (2006). Absorption and metabolism of conjugated α-linolenic acid given as free fatty acids or triacylglycerols in rats. Nutr Metab.

[B46] Mir PS, Mir Z, McAllister TA, Morgan Jones SD, He ML, Aalhus JL, Jeremiah LE, Goonewardene LA, Weselake RJ (2003). Effect of sunflower oil and vitamin E on beef cattle performance and quality, composition and oxidative stability of beef. Can J Anim Sci.

[B47] Averette Gatlin L, See MT, Hansen JA, Sutton D, Odle J (2002). The effects of dietary fat sources, levels, and feeding intervals on pork fatty acid composition. J Anim Sci.

[B48] Jing X, Nakamura MT, Cho HP, Clarke SD (1999). Sterol regulatory element binding protein-1 expression is suppressed by dietary polyunsaturated fatty acids. J Biol Chem.

[B49] Fernandez ML, Wood RJ, Conn PM (2008). Gunea pigs as models for human cholesterol and lipoprotein metabolism. Sourcebook of Models for Biomedical Research.

